# Exceptional population genomic homogeneity in the black brittle star *Ophiocomina nigra* (Ophiuroidea, Echinodermata) along the Atlantic-Mediterranean coast

**DOI:** 10.1038/s41598-023-39584-7

**Published:** 2023-07-31

**Authors:** Carlos Leiva, Laia Pérez-Sorribes, Sara González-Delgado, Sandra Ortiz, Owen S. Wangensteen, Rocío Pérez-Portela

**Affiliations:** 1 Departament de Biologia Evolutiva, Facultat de Biologia, Ecologia i Ciències Ambientals, Universitat de Barcelona (UB), Av. Diagonal 643, 08028 Barcelona, Spain; 2 University of Guam Marine Laboratory, 303 University Drive, Mangilao, GU 96923 USA; 3 Institut de Recerca de la Biodiversitat (IRBio), Universitat de Barcelona (UB), Barcelona, Spain; 4 Departamento de Biología Animal, Edafología y Geología, Facultad de Ciencias, Universidad de la Laguna, Canary Islands, Spain

**Keywords:** Population genetics, Zoology, Evolutionary genetics, Ecological genetics, Marine biology

## Abstract

The Atlantic-Mediterranean marine transition is characterised by strong oceanographic barriers and steep environmental gradients that generally result in connectivity breaks between populations from both basins and may lead to local adaptation. Here, we performed a population genomic study of the black brittle star, *Ophiocomina nigra*, covering most of its distribution range along the Atlantic-Mediterranean region. Interestingly, *O. nigra* is extremely variable in its coloration, with individuals ranging from black to yellow-orange, and different colour morphs inhabiting different depths and habitats. In this work, we used a fragment of the mitochondrial *COI* gene and 2,374 genome-wide ddRADseq-derived SNPs to explore: (a) whether the different colour morphs of *O. nigra* represent different evolutionary units; (b) the disruptive effects of major oceanographic fronts on its population structure; and (c) genomic signals of local adaptation to divergent environments. Our results revealed exceptional population homogeneity, barely affected by oceanographic fronts, with no signals of local adaptation nor genetic differentiation between colour morphs. This remarkable panmixia likely results from a long pelagic larval duration, a large effective population size and recent demographic expansions. Our study unveils an extraordinary phenotypic plasticity in *O. nigra*, opening further research questions on the ecological and molecular mechanisms underpinning coloration in Ophiuroidea.

## Introduction

The Atlantic-Mediterranean biogeographic marine region encompasses a wide range of subtropical, temperate and even subarctic marine areas that also includes several environmental transition zones and oceanographic fronts^1–4^. One of these relevant oceanographic transitions is the English Channel, which brings the southern Atlantic water mass into contact with the colder and shallower North Sea^5,6^. In the southern European area, the Strait of Gibraltar and the Almeria-Oran front define two oceanographic breaks, due to the strong and well-defined marine circulation system across this area, that separate the Atlantic and the Mediterranean basins^6,7^. These two barriers delimit the Alboran Sea, a mixing-water zone between the warmer and saltier Mediterranean Sea and the lower-salinity and colder Atlantic Ocean^8^. Depending on the species, these marine barriers have different effects on the connectivity and gene flow patterns between Atlantic and Mediterranean populations^6,7,9–14^, thus providing different disruptive effects of these two oceanographic barriers^3,9,10,12,13,15–18^. Genetic diversity patterns highlighted that large- and short-scale oceanographic circulation interplays with each species life history traits and its evolutionary history to determine its divergence level^3,6,19,20^. Therefore, it is mandatory to obtain genetic data from a wide range of species displaying a variety of biological traits and evolutionary histories, to extract general conclusions about the effects of oceanographic breaks on population connectivity, as well as the relative importance of each factor involved in the divergence patterns.

Although an array of studies on population genetics, phylogeography and speciation have been performed along the Atlantic-Mediterranean coast^9–17,21–24^, some animal groups, such as the ophiuroids (Ophiouroidea, Echinodermata) have been less studied. Ophiuroids, commonly known as brittle stars, are the most diverse echinoderm class, with important ecological roles in marine benthic food webs that occupy a large range of ecological and trophic niches in all oceans around the world^25–27^. To date, only a few studies on genetic connectivity and structure of ophiuroid populations along the European coast have been performed with nuclear markers^28–30^, whereas most studies were exclusively based on mitochondrial DNA^18,31^. These studies showed contrasting patterns of population divergence that were largely influenced by the reproductive strategy of the studied species (brooding *versus* non-brooding species), among other biological factors. Additionally, ophiuroids usually present cryptic species complexes^18,27,29,30,32–35^, and several cryptic species have been reported along the European Atlantic and Mediterranean coast for the genus *Ophioderma* (*O. longicauda* complex^30,33–35^, *Ophiothrix*^18,31^, and *Amphipholis* (*A. squamata* species complex^28,29^). Cases of overlooked diversity have also been reported, where different species had been mistakenly considered colour phases of the same species^32^, due to the generally large morphological plasticity found in this marine invertebrate group^18,36,37^.

Despite their high diversity and ecological relevance, genomic research on brittle stars is still on its infancy compared to other echinoderm classes. For instance, only three out of the 45 echinoderm genome assemblies available on NCBI are from ophiuroid species (https://www.ncbi.nlm.nih.gov/assembly, checked in February 2023). This general lack of genomic information prevents the implementation of genomic approaches that rely on reference genomes, which have remarkable applications on population genomics, evolution and conservation^38,39^. Nevertheless, reduced representation library techniques, such as restriction-site associated DNA sequencing (RADseq), enable the isolation of thousands of nuclear markers for population genomic analyses of non-model organisms^40^. Indeed, RADseq techniques have been successfully applied to a wide variety of non-model marine invertebrate species in absence of reference genomes: from sponges^41^ to molluscs^42^. In line with the lack of genomic resources for Ophiuroidea, there are only three studies using RADseq techniques on brittle stars, all of them focused on Antarctic species^43–45^.

In this work, we carry out a phylogeographic and population genomic study of the brittle star *Ophiocomina nigra*, for which different biological traits such as the feeding and social behaviour^46,47^, bioluminescence activity^48^, and secretory systems^49,50^ have been explored. *O. nigra* presents a wide distribution including the Mediterranean Sea and the Northeastern Atlantic Ocean, from the Azores Islands to Norway^37,47^, from shallow waters to relatively deep habitats (400 m). It is a slow-growing species that lives up to 14 years, and juveniles tend to settle far from adults^51^. *O. nigra* has a planktotrophic larva that remains in the water column for several months before its settlement, and therefore, with a potentially high dispersal ability^47^. Both field observations and laboratory experiments determined that *O. nigra* forms low-density and regular dispersed patches on the marine floor in the English Channel, where individuals avoid contact with each other, a social behaviour that largely differs from other co-occurring brittle stars such as *Ophiothrix fragilis*. However, *O. nigra* aggregations with densities over 100 individuals·m^−2^ might be induced by environmental disturbances or under presence of food^47^.

Interestingly, *O. nigra* presents an impressive colour variation, with individuals ranging from black to yellow-orange, and some organisms also presenting reddish hues^52^. In fact, dark and light colour morphs were once considered different species: the light-coloured morph was initially described as *Ophiocoma raschi* (Sars, 1871), but was later synonymised with *O. nigra*^53^. In the southern coast of Great Britain, the distribution of colour morphs has been related to depth, with darker colour morphs appearing more often at shallower areas and light-coloured individuals becoming more common in deeper waters^52^. Coloration of *O. nigra* depends on the oxidation state of a melanin pigment present in specialised melanin-containing cells of the integument, the melanocytes, which might be regulated by fluorescent pigments and an enzyme system controlling melanogenesis^54^. These differences in coloration, habitat preference and physiology could be due to phenotypic plasticity of a single species or, contrarily, they may indicate that they are in fact two different species, hypotheses that have not hitherto been contrasted from a genetic perspective.

In the present study, we sequenced a mitochondrial marker and isolated thousands of nuclear markers using a RADseq approach, in order to achieve the following objectives: (a) testing whether the two colour morphs of *O. nigra* are underpinned by genetic differences, and hence they should be considered different evolutionary units within a cryptic species complex; (b) assessing the genetic diversity, demographic history and population structure of *O. nigra*, focusing on the effects that multiple oceanographic barriers may have on its population connectivity; and c) looking for genomic signals of local adaptation to different environments. Our first hypothesis is that the two colour morphs of *O. nigra* may correspond to two cryptic species, as cryptic speciation is common in Ophiuroidea (e.g.^18,34^). Also, we anticipate high differentiation between distant localities due to the strong oceanographic barriers present in our study area. And finally, we expect to find genomic signals of local adaptation due to the contrasting habitats and sharp environmental gradients present in our sampling area.

## Results

**Figure 1:**
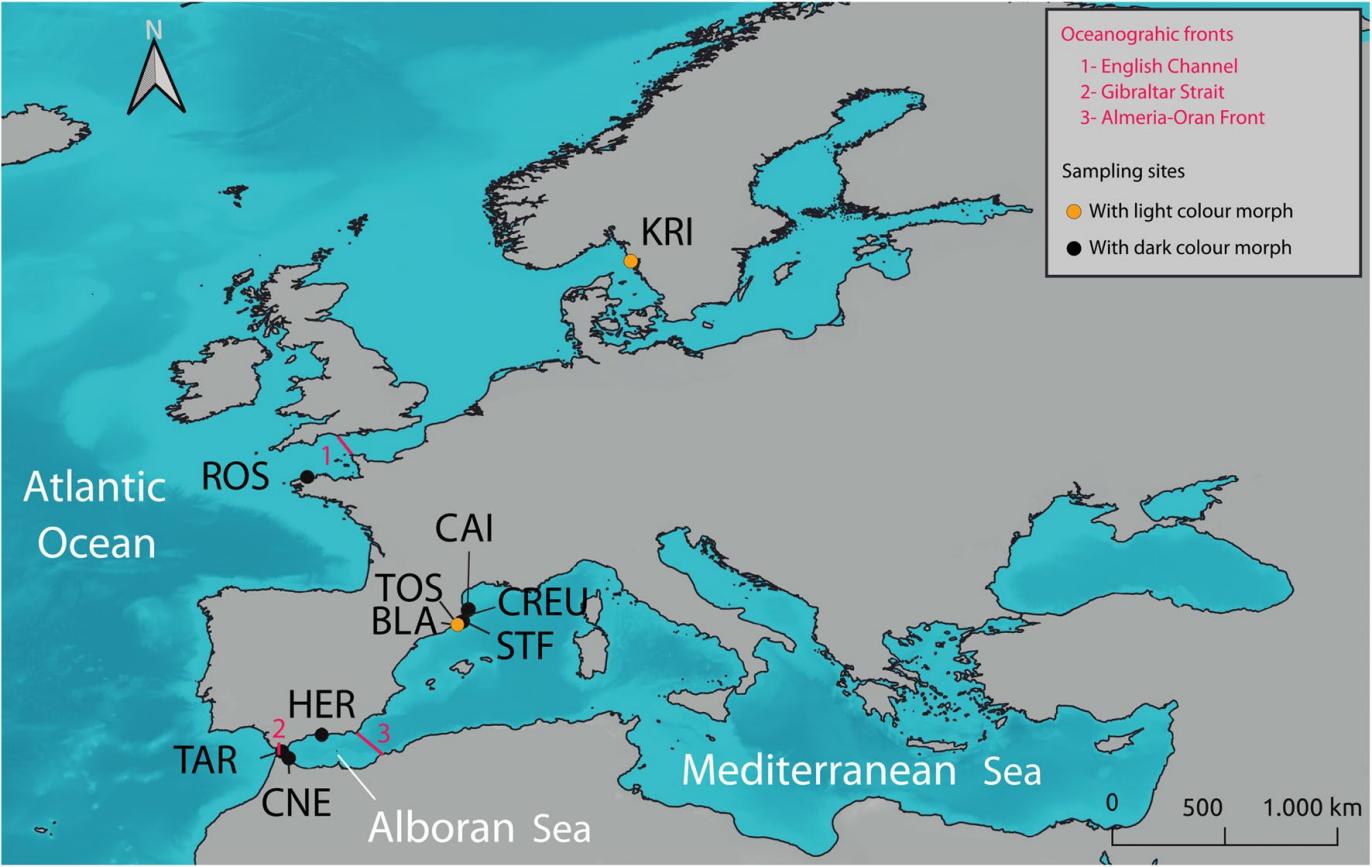
Map showing the study area and sampling localities. Major oceanographic areas are indicated (Atlantic Ocean; Alboran Sea; Mediterranean Sea) and major oceanographic fronts are represented by numbers (1, 2 and 3). Sampling stations are coded following Table 1: Es Caials, CAI; Cap de Creus, CREU; Sant Feliu de Guíxols, STF; Tossa de Mar, TOS; Blanes, BLA; La Herradura, HER; Cabo Negro, CNE; Tarifa, TAR; Roscoff, ROS; Kristineberg, KRI. Black points indicate localities where individuals collected belonged to the dark colour morph and orange points represent localities where individuals collected belonged to the light colour morph. Map was created in QGIS v.3.16.3^94^.

We collected 192 *Ophiocomina nigra* individuals from 10 localities in the Northwestern Mediterranean Sea, the Alboran Sea, and the Northeastern Atlantic Ocean (see Fig. 1 and Table 1). We identified 31 individuals that belonged to the light colour morph and 161 specimens that belonged to the dark colour morph. All individuals collected from Kristineberg (KRI; in Sweden) and from Blanes (BLA; in the NW Mediterranean, at 100–150 m depth) were those identified as belonging to the light colour morph. A fragment of 803 bp of the cytochrome *c* oxidase subunit I (*COI*) was amplified and sequenced for all organisms.

**Table 1:** Collection details for all sampling sites indicating region and sampling locality, code, coordinates, depth (in meters) and colour morph of the individuals collected in that locality.

Region/ locality	Code	Coordinates	Depth (m)	Colour morph
NW Mediterranean				
Es Caials, Spain	CAI	42°17′07.4″N 3°17′47.3″E	5–10	Dark
Cap de Creus, Spain	CREU	42°19′17.3″N 3°19′43.7″E	5–20	Dark
Sant Feliu de Guíxols, Spain	STF	41°46′15.5″N 3°02′47.8″E	5–20	Dark
Tossa de Mar, Spain	TOS	41°43′19.6″N 2°56′23.7″E	5–20	Dark
Blanes, Spain	BLA	41°37′36.0″N 2°46′42.0″E	100–150	Light
Alboran Sea				
La Herradura, Spain	HER	36°43′15.8″N 3°43′42.6″W	5–10	Dark
Cabo Negro, Morocco	CNE	35°41′04.7″N 5°18′25.2″W	5–10	Dark
Tarifa, Spain	TAR	36°00′10.3″N 5°36′24.4″W	5–10	Dark
Atlantic Ocean				
Roscoff, France	ROS	48°44′03.1″N 3°59′27.4″W	5–10	Dark
Kristineberg, Sweden	KRI	58°15′03.9″N 11°26′57.5″E	5–10	Light

From these 192 individuals, we sub-sampled 144 organisms that were used for *Single Nucleotide Polymorphisms* (SNP) isolation using double digest restriction-site associated DNA sequencing (ddRADseq). After filtering the Illumina reads, we obtained a total of *ca*. 272 M reads, and retained 109 individuals with an average of *ca*. 1.9 M reads per individual. The final dataset included 2,374 neutral SNPs and 109 individuals, containing 30.46% of missing data and an average depth of 9.6 reads per SNP per sample.

We detected a total of 124 outlier SNPs using Arlequin, as potential candidates under positive selection. However, no SNP was identified as under selection using Bayescan. Therefore, since we did not find common SNPs between the two methods, all SNPs were considered as neutral for subsequent population genomic analyses.

### Genetic diversity in *Ophiocomina nigra*

For the *COI* dataset, haplotype number, nucleotide diversity (π) and haplotype diversity (Hd) values were high in all *O. nigra* localities (Table 2). Nucleotide diversity ranged from 0.008 in Cap de Creus (CREU) to 0.017 in Tossa (TOS), with a value of 0.014 for the whole dataset. Haplotype diversity values were close to 1 in all localities, with a value of 0.989 for the whole dataset. From the total of 192 individuals sequenced, we identified 125 different *COI* haplotypes, 99 of them being private. Considering three major marine regions (Fig. 1), 17 private haplotypes belonged to the Atlantic localities, 48 to the Alboran Sea, and 40 to the NW Mediterranean localities. Genetic diversity parameters per locality, colour morph, and geographical region (Atlantic Ocean, Alboran Sea and NW Mediterranean) are shown in Table 2.

**Table 2:** Population genetic diversity indices for the *COI* dataset including the number of individuals sequenced (N), number of *COI* haplotypes (h), number of private haplotypes (ph), nucleotide diversity (π) (± SD), haplotype diversity (Hd) (± SD), and the three neutrality tests performed: Tajima’s D, Fu’s Fs and R2 (* p < 0.05). Data are shown by locality, colour morph and region.

	N	h	ph	π	Hd	Tajima's D	Fu’s Fs	R2
Locality								
CAI	21	15	4	0.015 (± 8.5 × 10^−4^)	0.952 (± 0.032)	−0.31	−0.241	0.121
CREU	4	4	2	0.008 (± 0.00157)	1 (± 0.177)	−0.605	−0.615	0.06
STF	28	27	20	0.016 (± 9.2 × 10^−4^)	0.997 (± 0.01)	−1.457	−2.486	0.066
TOS	5	5	2	0.017 (± 0.00312)	1 (± 0.126)	−0.26	−0.235	0.115
BLA	21	19	9	0.014 (± 8.7 × 10^−4^)	0.990 (± 0.018)	−0.351	0.16	0.121
HER	34	31	23	0.015 (± 7.3 × 10^−4^)	0.993 (± 0.01)	−0.954	−1.158	0.086
CNE	9	9	4	0.015 (± 0.00156)	1 (± 0.052)	0.156	0.274	0.17
TAR	40	32	20	0.014 (± 6.7 × 10^−4^)	0.985 (± 0.01)	−0.882	−0.594	0.091
ROS	20	19	13	0.014 (± 0.00104)	0.995 (± 0.018)	−0.837	−0.838	0.103
KRI	10	10	4	0.011 (± 0.00134)	1 (± 0.045)	0.326	0.01	0.154
Colour morph								
Dark	31	29	13	0.015 (± 8.1 × 10^−4^)	0.996 (± 0.009)	−0.607	−0.133	0.102
Light	161	112	97	0.015 (± 3.1 × 10^−4^)	0.9897 (± 0.003)	−1.439	−2.687	0.05
Region								
NW Mediterranean	79	58	40	0.015 (± 4.3 × 10^−4^)	0.984 (± 0.007)	−1.275	−2.264	0.062
Alboran Sea	83	65	48	0.015 (± 4.4 × 10^−4^)	0.991 (± 0.004)	−1.118	−1.332	0.07
Atlantic Ocean	30	28	17	0.013 (± 8.7 × 10^−4^)	0.995 (± 0.010)	−0.919	−0.995	0.093
Total	192	125		0.015 (± 2.9 × 10^−4^)	0.989 (± 0.003)	−1.417	−2.715 *	0.049

Population genomic statistics for the SNP dataset are shown in Table 3. Allele number and allele effective number were similar across the sampling stations, ranging from 1.640 in Kristineberg (KRI) to 1.956 in Tarifa (TAR) for the number of alleles, and from 1.337 in Kristineberg (KRI) to 1.376 in Tarifa (TAR) for the effective number of alleles. Genetic diversity, measured as observed heterozygosity (Ho) ranged from 0.203 in Es Caials (CAI) to 0.233 in Cabo Negro (CNE), with a value of 0.222 for the entire dataset, and expected heterozygosity (He), ranged from 0.236 in Es Caials (CAI) to 0.253 in Cabo Negro (CNE), with a value of 0.246 for the whole dataset. Observed heterozygosity was lower than the expected value in all sites and for the whole dataset. Inbreeding coefficients (F_IS_) presented low values in all sampling sites, ranging from 0.079 in Cabo Negro (CNE) to 0.138 in Es Caials (CAI), with a total value of 0.099. All populations were deviated from the Hardy–Weinberg equilibrium as demonstrated by their significant values.

**Table 3:** Population genetic diversity indices for the ddRADseq-derived SNP dataset including the number of individuals retained after filtering (Nf), number of alleles (An), effective number of alleles (A_Eff n), observed heterozygosity (Ho), expected heterozygosity (He), and inbreeding index (F_IS_) with the Hardy–Weinberg Equilibrium p-value (**p-value < 0.01). We also included the three neutrality tests performed: Tajima’s D, Fu’s Fs and R2. Data are shown by locality, colour morph and region.

	Nf	An	A_Eff n	Ho	He	F_IS_	Tajima's D	Fu's Fs	R2
Locality									
CAI	12	1.728	1.338	0.203	0.236	0.138**	−0.708	−0.611	0.175
STF	14	1.889	1.369	0.225	0.249	0.095**	−0.952	−1.641	0.119
BLA	15	1.841	1.357	0.216	0.242	0.109**	−0.935	−1.394	0.132
HER	19	1.925	1.367	0.222	0.246	0.097**	−1.115	−2.078	0.105
CNE	10	1.818	1.372	0.233	0.253	0.079**	−0.802	−0.951	0.15
TAR	21	1.956	1.376	0.229	0.252	0.089**	−1.16	−2.712	0.092
ROS	12	1.776	1.352	0.216	0.241	0.103**	−0.747	−0.8	0.156
KRI	6	1.64	1.337	0.215	0.241	0.106**	−0.473	−0.224	0.22
Colour morph									
Dark	21	1.921	1.36	0.216	0.242	0.109**	−1.115	−2.244	0.103
Light	88	2	1.372	0.223	0.247	0.097**	−1.9	−12.431	0.032
Region									
NW Mediterranean	41	1.989	1.365	0.217	0.243	0.108**	−1.498	−4.905	0.063
Alboran Sea	50	1.998	1.376	0.227	0.25	0.09**	−1.664	−7.123	0.05
Atlantic Ocean	18	1.88	1.356	0.215	0.241	0.105**	−0.998	−1.68	0.115
Total	109	2	1.371	0.222	0.246	0.099**	−1.971	−15.4	0.027

### Population connectivity and structure

#### COI results

**Figure 2:**
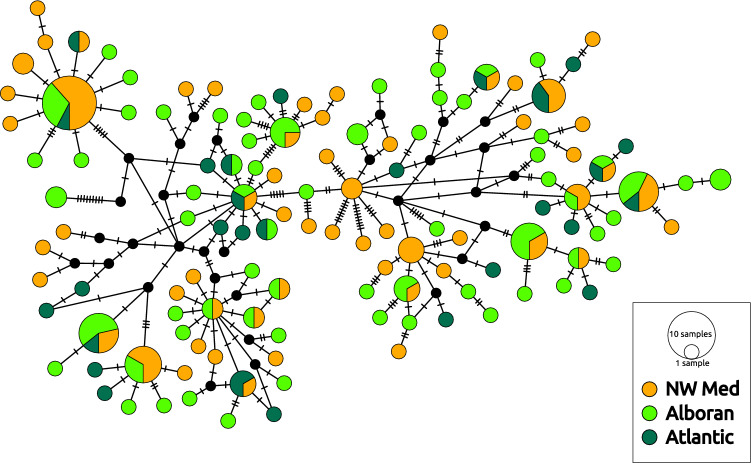
Unrooted haplotype network for the mitochondrial *COI* marker of *Ophiocomina nigra*. Circle size is proportional to the number of individuals sharing each haplotype. Colours represent regions: NW Mediterranean (NW Med); Alboran Sea (Alboran); Atlantic Ocean (Atlantic), and the black small circles represent missing haplotypes. Crossed black lines represent mutational steps between haplotypes.

The *COI* haplotype network is shown in Fig. 2 (haplotype frequencies per population available in Mendeley Data at https://doi.org/10.17632/5m6v5sm5f6.1). It reveals deep diverse bush-like genealogies, with twenty shared haplotypes and a large number of singletons. The shared haplotypes suggest population homogeneity and high population connectivity, with haplotypes shared among individuals from the three different regions studied (Fig. 2). Interestingly, no structure was either detected between colour morphs, pointing to phenotypic plasticity as the mechanism explaining the environmental segregation of colour morphs in *O. nigra*. In agreement with these results, the pairwise ϕ_ST_ table (Supplementary Material SM1) showed low and non-significant values for all pairwise comparisons, ranging from 0.001 to 0.032, revealing low differentiation between sampling stations across our study area. Analyses of molecular variance (AMOVAs) showed that there was no significant genetic differentiation by region or colour morph in the *COI* dataset (Supplementary material SM2), in agreement with the previously exposed results showing the high genetic homogeneity of *O. nigra* among sampling stations and regions.

#### ddRADseq-derived SNP results

**Figure 3:**
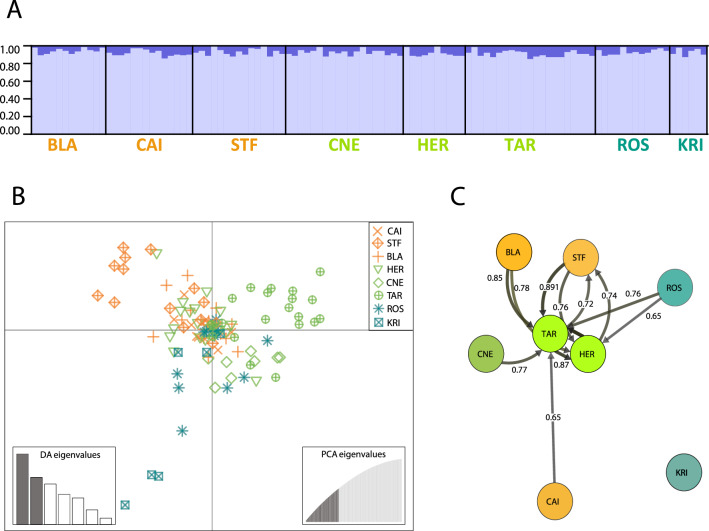
Population genomic results for the 2,374 neutral SNP dataset. (**A**) STRUCTURE results for K = 2 (see Delta K plot in Supplementary Material SM4). (**B**) Two-dimensional representation of the DAPC results, using sampling stations as a priori grouping. Points represent different individuals and point patterns represent sampling sites. The DA and PCA sub-graphs represent the number of discriminant functions and principal components retained for the analysis, respectively. (**C**) *G*_ST_ migration network results showing relative migration values higher than 0.65 (see complete *G*_ST_ migration network in Supplementary Material SM8). The circles represent the sampling sites and the numbers on the arrows show the relative migration among sampling sites. For (**B**) and (**C**) colours represent the three different regions: orange = NW Mediterranean Sea; green = Alboran Sea; and turquoise = Atlantic Ocean.

Population structure results for the ddRADseq-derived SNP dataset also showed high levels of homogeneity across regions. Pairwise F_ST_ table (Supplementary Material SM1) showed low to moderate values of differentiation, ranging from 0 to 0.168. Only two comparisons were significantly different from zero (p-value after correction < 0.05: Cabo Negro (CNE) – Tarifa (TAR) and Cabo Negro (CNE) – La Herradura (HER), despite the short geographic distance among these three locations). AMOVA results for the SNP dataset mirrored the results obtained with the mitochondrial *COI* dataset, and no significant genetic differentiation was found either by region or by colour morph (Supplementary material SM3). Results from STRUCTURE^55^ also suggested genetic homogeneity along the studied geographical area with all individuals belonging to one main cluster (Fig. 3A and Supplementary material SM5), despite the most likely number of genetic clusters were two according to the Delta K or four according to the Ln posterior probabilities (Supplementary Material SM4 and SM5). The Bayesian Information Criterion (BIC) results, as implemented in the Discriminant Analysis of Principal Components (DAPC)^56^, showed that one cluster was again the optimal number to describe our data (Supplementary Material SM6). However, a subtle substructure emerged when sampling site information was used as *a prior* for the DAPC (Fig. 3B), with some differentiation between the three regions. The complete G_ST_ migration network is shown in Supplementary Material SM8. The highest relative migration values (> 0.65) are represented in Fig. 3C, which indicated an isolation of Kristineberg (KRI) from the rest of our study area. Locations of the Alboran Sea, particularly Tarifa (TAR) and La Herradura (HER), appeared as central in the migration network, presenting the highest migration values and mostly acting as sink populations (Fig. 3C).

### Demographic history of *Ophiocomina nigra*

Neutrality tests and demographic history analyses based on both *COI* and ddRADseq-derived SNP datasets demonstrated recent demographic expansions in *O. nigra*.

Neutrality tests for the *COI* dataset are shown by locality, colour morph and region in Table 2. Tajima’s D for sampling sites ranged from −1.457 in Sant Feliu de Guíxols (STF) to 0.326 in Kristineberg (KRI), with a value of −1.417 when analysing all samples together. Fu and Li’s F values for sampling stations ranged from −2.486 in Sant Feliu de Guíxols (STF) to 0.16 in Blanes (BLA), with a significant value of −2.715 for the whole dataset. Ramos-Onsin and Rozas’s R2 statistic ranged from 0.06 in Cap de Creus (CREU) to 0.17 in Cabo Negro (CNE), presenting a value of 0.049 when all samples were analysed together.

**Figure 4:**
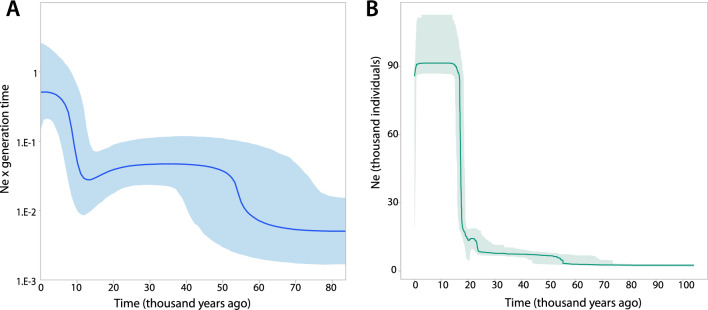
Demographic history analyses for *Ophiocomina nigra.* (**A**) Bayesian Skyline Plot results for the *COI* dataset. Time is represented in thousand years ago, the dark blue line illustrates mean size estimations and the blue shaded area shows the 95% confidence interval. (**B**) Stairway Plot 2 results for the ddRADseq-derived SNP dataset. The turquoise line illustrates the median estimate of the effective population size through time in thousand years ago. The shaded area represents the 75% confidence interval.

The Mismatch distribution indicated that the *COI* data did not fit an unimodal expansion model, as shown by the significant values of the *r* statistic (r = 0.0027, *p* = 0.001), but the bimodal distribution on the observed pairwise differences may correspond to two consecutive expansion events (Supplementary Material SM7). The coalescent-based Bayesian Skyline Plot (BSP) for the *COI* dataset supported the existence of two population growth events, the most ancient one occurring between 50,000–60,000 years ago, and the most recent starting at 10,000 years ago until the present days (Fig. 4A).

Neutrality tests for the ddRAD data are shown by locality, colour morph and region (Table 3). Tajima's D values for sampling stations ranged from −1.16 in Tarifa (TAR) to −0.476 in Kristineberg (KRI), with an overall value of −1.971. Fu’s Fs values for sampling stations ranged from −2.712 in Tarifa (TAR) to −0.224 in Kristineberg (KRI), with a value of −15.4 for the whole dataset. Ramos-Onsin and Rozas’s R2 values for sampling stations ranged from 0.092 in Tarifa (TAR) to 0.22 in Kristineberg (KRI), with an overall value of 0.027.

Stairway Plot 2^57^ results using the folded site frequency spectrum derived from the SNP dataset revealed the particular timing when the most recent population expansion occurred (Fig. 4B). This recent expansion started at around 50,000 years ago, in agreement with the BSP results, and presented a very steep population growth between 20,000 and 15,000 years ago.

## Discussion

The study here presented is the first exploring the population genomic structure, phylogeography, and demographic history patterns of the brittle star *Ophiocomina nigra*. To date, genome-wide scans for population genomics of ophiuroids only focused on three Antarctic species^43,44^. Although *O. nigra* has been deemed as uncommon in the Mediterranean Sea^37,52^, we report here populations in our Mediterranean sampling sites. We suggest that this is not due to a recent expansion of this species into the Mediterranean Sea in the last 80 years, as we did not find strong genetic signals of founder effects. We consider that the Mediterranean populations were overlooked in the past.

Our population genomic results, based on 2,374 ddRADseq-derived SNPs and a fragment of the mitochondrial *COI* marker, revealed population homogeneity across the whole study area, from the cold waters of the Northeastern Atlantic Ocean to the Mediterranean Sea. Our results demonstrated that the two different colour morphs present in *O. nigra* correspond to a single evolutionary unit, as no genetic divergence was observed between them. These results contrast with the high number of cryptic speciation events detected in other brittle stars in the same geographical area, such as *Ophiothrix* spp^18,31^, *Ophioderma longicauda* and *Amphipholis squamata* species complexes^30,34^. Hence, our results point to a large phenotypic plasticity as the driver of the morphological differences found between *O. nigra* colour morphs. As previously suggested, colour variation in *O. nigra* may depend on the specific environment where an organism lives^52^. In general, individuals inhabiting shallower depths tend to exhibit darker colours, whereas in deeper habitats, the individuals have lighter tones^52^. These differences in pigmentation have been associated with the higher melanin production that individuals from shallower habitats present as a form of sun protection^52,58^. Another factor that has been suggested to affect body coloration in *O. nigra* is the substrate characteristics^52^. However, we cannot test this hypothesis because no details on the substrate type were collected during our sampling.

Despite this morphological variation within the species, and the large geographical range explored, we found general population homogeneity. We detected high levels of genetic diversity for both the mitochondrial *COI* fragment and the nuclear SNPs, but no population divergence (see Figs. 1, 2 and 3). The major oceanographic fronts included in our study area, the English Channel, the Gibraltar Strait, and the Almería-Oran front had a minor disruptive effect on the population structure of *O. nigra*, a result that contrasts with those observed in most coastal benthic species (e.g.^3,7,18^), including other brittle stars^18^. Such a pattern of panmixia, or low genetic divergence, between the Mediterranean and Atlantic basins, or within the Mediterranean sub-basins is relatively common in echinoderms with large dispersal potential^15,21,59^ and in those with a predominance of asexual reproduction^9,17^. Nevertheless, the absence of divergence found in *O. nigra* between the Northeast Atlantic and the Mediterranean is an exceptional pattern even for species with large dispersal potential^12,14,15,21,59^. Past oceanographic processes across the Atlantic-Mediterranean transition^6^, and the current disruptive effect of major oceanographic circulation across the Gibraltar Strait and the Almería-Oran front, have generated a large variety of genomic patterns in marine invertebrates, but most of them are characterised by a major divergence between these two main basins^12,14,16^, although exceptions also exist^31^. Interestingly, within the *Ophiothrix* species complex, *Ophiothrix* sp. II displayed a lack of genetic divergence similar to that obtained in *O. nigra*, with genetic homogeneity along the Atlantic-Mediterranean transition^18,31^. However, for *Ophiothrix* sp. II the sampling area was restricted to the Iberian Peninsula and only two mitochondrial markers were studied^18^.

The lack of significant differentiation between distant geographical areas in *O. nigra* may result from the combination of different biological features, demographic and historical events, including a large dispersal potential of the larva, large effective population size, and past demographic expansions^14,18,31^. The planktotrophic larva of *O. nigra* remains in the water column for several months avoiding settlement near adults^51^, and hence can be dispersed hundreds of kilometres by marine currents^47^. A long dispersal potential during early life stages, which interacts with oceanographic circulation, significantly boosts population connectivity^60^. Additionally, the low-density and regularly dispersed patches formed by *O. nigra* across large spatial areas^47^ may favour connectivity along distant sites.

The population homogeneity found in *O. nigra* could be also the result of the recent demographic expansions that we identified from both *COI* and SNPs datasets. Our demographic history analyses identified a recent effective population expansion consisting in two growth periods, likely related to expansions during Pleistocene interglacial periods (e.g.^12,18^). The ancient growth period was recovered from both the *COI* BSP and the ddRADseq Stairway Plot 2 results, and was dated between 50,000 and 60,000 years ago, similar to that dated for *Ophiothrix* spp^18^ and some other Mediterranean species^61^. The most recent one was also identified by both datasets, and pointed to a tenfold increase of *O. nigra* populations between 10,000 and 20,000 years ago (see Fig. 4). This coincides with the deglaciation period that followed the Last Glacial Maximum (LGM)^62^, and is the most common post-LGM population expansion pattern found in other marine invertebrates (see an example in^61^). This recent demographic expansion could have partially erased previous population differentiation in *O. nigra*, favouring the current genetic homogeneity found in both datasets. Additionally, a large effective population size reduces genetic drift effects^63^, reducing genetic differentiation between populations and increasing genetic homogeneity.

Despite the general pattern of population homogeneity found in *O. nigra*, the presence of sub-regional private haplotypes in the *COI* marker suggested a certain degree of genetic substructuring in major geographical areas. In agreement with these results, migration analyses based on ddRADseq-derived SNPs showed that gene flow is not symmetric and homogeneous over our study area. The migration network (Fig. 3C) showed that Kristineberg (KRI) was isolated from all other sites, whereas Roscoff (ROS) and the NW Mediterranean sites were all connected to two sites from the Alboran Sea, Tarifa (TAR) and La Herradura (HER). This asymmetric migration pattern reveals a disruptive effect of the Gibraltar Strait or the Almería-Oran front on gene flow. Therefore, the role of the Alboran Sea as a transitional area connecting these two major basins, the Mediterranean Sea and the Atlantic Ocean^3,9,15,22^, is also found in *O. nigra*.

Finally, against our initial expectations, we did not find genomic signals of selection and/or local adaptation in *O. nigra* across our studied area, which covers important temperature and salinity clines. This result in *O. nigra* contrasts with those from other echinoderms that displayed signals of selection and local adaptation to temperature and salinity^15,21^, even under high levels of gene flow. However, due to the small proportion of the genome that is covered by RADseq approaches, strong selection associated with environmental variables might be affecting other genomic regions that remained uncovered in this study^64^.

Overall, our study shows an exceptionally high genetic homogeneity in *O. nigra*, barely disturbed by the disruptive effects of the English Channel, the Gibraltar Strait and the Almería-Oran front. This genetic homogeneity likely results from a combination of a long pelagic larval duration, a large effective population size, and recent demographic expansions. Our study also demonstrates that the different colour morphs present in *O. nigra* are the result of high phenotypic plasticity of the species, opening new questions for further research investigating the ecological and molecular mechanisms behind this intriguing plasticity.

## Methods

### Sampling, DNA extraction and sequencing

A total of 192 specimens of *Ophiocomina nigra* were sampled across the natural distribution range of the species, from 10 different localities throughout the Atlantic-Mediterranean region between 2014 and 2017 (see details in Fig. 1 and Table 1). Specimens were collected between 5–20 m depth by SCUBA diving for most localities, except for those from Blanes (BLA, NW Mediterranean), which were collected by local fishermen trawling between 100–150 m depth. All samples were preserved in absolute ethanol at room temperature until further processing.

Among all samples, we identified the two colour morphs previously described for this species and likely associated with depth (see Table 3 in^19^). See collection details in Table 1.

#### Ethics statement

No endangered or protected species were involved in this study. Authors possessed the required permits to collect echinoderm samples for research.

#### Mitochondrial COI marker sequencing

Genomic DNA was extracted from ethanol-preserved tube feet using the REDExtract-N-Amp Tissue extraction kit protocol (Sigma-Aldrich, www.sigma.com). Specific primers for a fragment of the mitochondrial cytochrome *c* oxidase subunit I (*COI*) marker of *O. nigra* were designed using Primer v. 3. 0 (http://simgene.com/Primer3) from the available sequence in GenBank (Accession number FN562577.1). These newly designed primers were: Onig-F (5′-AGTCGGTGATTATTTTCTACAA-3′) and Onig-R (5′-AATTATCATTGTAGCAGCAGTG-3′). PCR conditions were as follows: initial denaturalization at 94 °C for 5 min, 40 cycles of 94 °C for 45 s, 50 °C for 45 s and 72 °C for 90 s, and a final extension of 72 °C for 5 min. The PCR-amplified products were bi-directionally sequenced at Macrogen Europe. Sequences were edited using BioEdit v. 7.0^65^ and aligned with ClustalW as implemented in Mega v. 6^66^.

#### ddRAD library preparation, sequencing and filtering

A subset of 144 specimens from eight representative *O. nigra* populations, was used to generate double digest restriction site associated DNA (ddRAD) libraries following^67^. High molecular weight DNA was re-extracted from preserved samples following a modified cetyltrimethylammoniumbromide (CTAB) protocol^68^. Briefly, tube feet tissue samples were digested overnight at 57 °C in CTAB buffer (2% w/v CTAB, 1.4 M NaCl, 20 mM EDTA, 100 mM Tris- HCl, pH 8), 2-mercaptoethanol and Proteinase K. DNA was subsequently isolated with 24:1 chloroform:isoamyl alcohol and precipitated with ice-cold ethanol. DNA integrity, purity, and quantity were checked in agarose electrophoresis gels, Nanodrop (Thermo Fisher), and using a Qubit DNA HS assay (Life Technologies), respectively. For ddRAD library preparation, we double digested 510 ng of DNA per sample using high-fidelity restriction enzymes EcoRI and MseI (New England Biolabs). Fragments per sample were purified with Agencourt AMPure beads (Beckham Coulter) before ligation with barcoded Illumina adapters. Samples with unique adapters were pooled, and each pool was size-selected (200 to 400 bp) using a Blue Pippin (Sage Science). Illumina multiplexing indices were added to each library during PCR amplification with a Phusion high-fidelity DNA polymerase kit (New England Biolabs), using a common forward primer (PCR1) and a reverse library-specific primer (indexed PCR2). Six libraries, with 24 individuals per library, were paired-end sequenced (150 bp) on an Illumina NovaSeq 6000 at the Novogene Core Facility, Cambridgeshire, UK.

Quality filtering, locus assembly and SNP calling were conducted using the Stacks pipeline v. 2.59^69^. RAD-tags (DNA fragments with the two appropriate restriction enzyme cut sites that were selected, amplified, and sequenced) were processed using process_radtags, where raw reads were quality-trimmed to remove low quality reads, reads with uncalled bases, and reads without a complete barcode or restriction cut site. After these filtering steps, we retained a total of 272,024,358 reads from the initial 307,074,382 raw reads, with an average of 1,889,058 reads per sample. Then, the core Stacks pipeline was run with optimised parameters following^70^ and^71^. Optimal parameter values were: m = 3, M = 4, and n = 4. The Stacks population module was used to conduct a first filtering of the data, retaining SNPs present in at least 50% of the individuals (r = 0.5) and with a minimum allele frequency higher than 0.05 (–min-maf 0.05). We also used the “–write-single-snp” option in order to retain only the first SNP from each RAD-tag to prevent the inclusion of physically linked loci in the dataset. We then filtered out individuals with less than 20% of the loci using the adegenet R package^72^. Then, we used the perl script “filter_hwe_by_pop.pl” from the dDocent pipeline^73^ with default parameters (-h 0.001, -c 0.25) to filter SNPs deviating from Hardy–Weinberg equilibrium within sampling stations.

The identification of putative SNPs under selection was performed using Arlequin v. 3.5^74^ and Bayescan v. 2.1^75^. Arlequin was run with default parameters using 100,000 coalescent simulations and 100 demes. Benjamini-Yekutieli FDR corrections were applied to 0.01 p-value. Bayescan was run with default parameters, using the -snp flag and a false discovery rate of 0.05 (FDR = 0.05). Only SNPs identified by both methods were considered as being under selection and removed from the neutral SNP dataset used for subsequent population genomic analyses.

### Population structure and genetic diversity analyses

#### Mitochondrial COI gene fragment

We calculated diversity indices for the *COI* data, including haplotype diversity (Hd), nucleotide diversity (π), the number of haplotypes for the different populations (H), and number of private haplotypes per sampling site, colour morph and region using DnaSP v. 5.10^76^. The software PopART^77^ was used to construct an un-rooted haplotype network using the TCS statistical parsimony network approach^78^.

Pairwise F_ST_ indices were estimated between sampling stations using Arlequin with 90,600 permutations to determine their associated *p*-values. We used a Benjamini-Yekutieli false discovery rate correction for multiple comparisons^79^ and an overall corrected 0.05 α-level. In order to test whether the groupings of samples by colour morph or by region explained a significant part of the total genetic variation we performed two analyses of molecular variance (AMOVA) in Arlequin.

#### Neutral ddRAD data

Indices of genetic diversity including mean number of alleles, expected heterozygosity (He), observed heterozygosity (Ho), inbreeding index (F_IS_), and the Hardy–Weinberg Equilibrium were estimated by sampling site, colour morph and region using GenoDive v. 3.05^80^.

Pairwise *F*_ST_ indices and AMOVAs were also estimated for the ddRADseq-derived SNP dataset using Arlequin as detailed above. Population structure was assessed using STRUCTURE v. 2.3.4^55^ and the discriminant analysis of principal components (DAPC)^56^ as implemented in the adegenet R package^72^. STRUCTURE was run using the admixture model for 500,000 MCMC iterations, with a burn-in of 50,000 iterations, setting the number of clusters (K) from 1 to 9, with 10 replicates per K. We used STRUCTURE HARVESTER^81^ to inspect the most likely number of K, and CLUMPAK^82^ to average individual’s membership coefficients across replicates and graphically represent STRUCTURE results. The DAPC was performed estimating the number of clusters with the find.clusters function in adegenet, using the optim.a.score function to optimise the number of retained PCs. Additionally, in order to identify gene flow patterns, a relative migration network analysis was conducted with the divMigrate function in the diveRsity R package^83,84^ using Nei’s *G*_ST_ method.

### Demographic history analyses

In order to unveil the evolutionary history of *Ophiocomina nigra* and infer how it might have affected the current genetic diversity distribution and population structure, we ran demographic history analyses for both *COI* and ddRAD data.

For both datasets, Tajima’s D and Ramos-Onsins and Rozas’ R2 neutrality tests were calculated for each sampling station, colour morph and region, and for the whole datasets using DnaSP v. 6.12^85^. In addition, Fu’s Fs and Fu and Li’s F neutrality tests were calculated for the ddRAD and the *COI* datasets, respectively, for each sampling site, colour morph and region, using DnaSP. We used the fasta file generated by the Stacks population module with the -fasta-samples option as the ddRAD data input file for DnaSP.

For the *COI* data, we calculated the pairwise mismatch distribution using DnaSP and performed a Bayesian Skyline Plot (BSP) using BEAUTi and BEAST v. 1.10.4^86^. For the pairwise mismatch distribution, the effective population size parameter (θ) was set to follow the Rogers & Harpending model^87^. In order to determine if the empirical data fitted an expansion model we calculated the raggedness (r) index^88^ using 10,000 permutations. Our priors for the BSP model included the implementation of the substitution model previously defined by jModelTest v. 2.1.10^89^ (HKY + I + G), a strict clock model, and the constant skyline model. As no molecular clock has been calibrated for ophiuroids, a mutation rate of 1.01% per nucleotide per million years was used for the *COI*, following other studies in echinoderms and the most recent estimations for this group^90^. Analyses were run for 500 million generations, with a sampling frequency of 50,000 generations, and a burning of 25% of the MCMC. The software Tracer v.1.7.2^91^ was used for assessing stationarity of the MCMC, effective sample sizes (ESSs > 200) and generating the evolution of effective population size under the skyline plot model, expressed as NeT (T = generation time) over time.

Additionally, we ran Stairway Plot 2^57^ using the ddRADseq-derived SNP dataset. Firstly, we obtained a new vcf file using the Stacks populations module only applying the -r 0.5 option. From this vcf file we obtained the folded Site Frequency Spectrum (SFS) using the easySFS software (https://github.com/isaacovercast/easySFS) with 114 projections. We prepared the blueprint file for Stairway Plot 2 using the following parameters: nseq = 114; L = 3,415,039 (from the populations module output); whether_folded = true; smallest_size_of_SFS_bin_used_for_estimation = 1 (to include singletons in the analysis); nrand = 28, 56, 84, 112; ninput = 200; mu = 9.4e−9 (nuclear mutation rate for other echinoderms^92^); year_per_generation = 7. Results were plotted using the ggplot2 R package^93^.

## Supplementary Information

Supplementary Information.

## Data Availability

*COI* sequences and Illumina raw reads for all individuals are available on NCBI GeneBank (Accession Numbers: OQ152818-OQ152942) and SRA database (BioProject ID PRJNA938024; Samples SAMN33423011-SAMN33423119), respectively.

## References

[1] Beaugrand G, Edwards M, Hélaouët P (2019). An ecological partition of the Atlantic Ocean and its adjacent seas. Prog. Oceanogr..

[2] Fernández V, Dietrich DE, Haney RL, Tintoré J (2005). Mesoscale, seasonal and interannual variability in the Mediterranean Sea using a numerical ocean model. Prog. Oceanogr..

[3] Pascual M, Rives B, Schunter C, Macpherson E (2017). Impact of life history traits on gene flow: A multispecies systematic review across oceanographic barriers in the Mediterranean Sea. PLoS ONE.

[4] Sutton TT (2017). A global biogeographic classification of the mesopelagic zone. Deep Sea Res. Part I.

[5] Ayata S-D, Lazure P, Thiébaut É (2010). How does the connectivity between populations mediate range limits of marine invertebrates? A case study of larval dispersal between the Bay of Biscay and the English Channel (North-East Atlantic). Prog. Oceanogr..

[6] Patarnello TOMA, Volckaert FAMJ, Castilho RITA (2007). Pillars of Hercules: Is the Atlantic-Mediterranean transition a phylogeographical break?. Mol. Ecol..

[7] El Ayari T, Trigui El Menif N, Hamer B, Cahill AE, Bierne N (2019). The hidden side of a major marine biogeographic boundary: A wide mosaic hybrid zone at the Atlantic-Mediterranean divide reveals the complex interaction between natural and genetic barriers in mussels. Heredity.

[8] El-Geziry T, Bryden I (2010). The circulation pattern in the Mediterranean Sea: Issues for modeller consideration. J. Oper. Oceanogr..

[9] Pérez-Portela R, Garcia-Cisneros A, Campos-Canet M, Palacín C (2021). Genetic homogeneity, lack of larvae recruitment, and clonality in absence of females across western Mediterranean populations of the starfish *Coscinasterias tenuispina*. Sci. Rep..

[10] Pérez-Portela R, Noyer C, Becerro M (2014). Genetic structure and diversity of the endangered bath sponge *Spongia lamella*. Aquat. Conserv..

[11] Perez-Portela R, Turon X (2008). Cryptic divergence and strong population structure in the colonial invertebrate *Pycnoclavella communis* (Ascidiacea) inferred from molecular data. Zoology.

[12] Perez-Portela R, Villamor A, Almada V (2010). Phylogeography of the sea star *Marthasterias glacialis* (Asteroidea, Echinodermata): Deep genetic divergence between mitochondrial lineages in the north-western Mediterranean. Mar. Biol..

[13] Pérez-Portela R (2019). Spatio-temporal patterns of genetic variation in *Arbacia lixula*, a thermophilous sea urchin in expansion in the Mediterranean. Heredity.

[14] Pérez-Portela R, Rius M, Villamor A (2017). Lineage splitting, secondary contacts and genetic admixture of a widely distributed marine invertebrate. J. Biogeogr..

[15] Carreras C (2021). The two sides of the Mediterranean: Population genomics of the Black Sea Urchin *Arbacia lixula* (Linnaeus, 1758) in a Warming Sea. Front. Mar. Sci..

[16] Garcia-Cisneros A, Palacín C, Ben Khadra Y, Pérez-Portela R (2016). Low genetic diversity and recent demographic expansion in the red starfish *Echinaster sepositus* (Retzius 1816). Sci. Rep..

[17] Garcia-Cisneros A (2018). Intraspecific genetic structure, divergence and high rates of clonality in an amphi**-**Atlantic starfish. Mol. Ecol..

[18] Taboada S, Pérez-Portela R (2016). Contrasted phylogeographic patterns on mitochondrial DNA of shallow and deep brittle stars across the Atlantic-Mediterranean area. Sci. Rep..

[19] Avise JC (1987). Intraspecific phylogeography: The mitocondrial DNA bridge between population genetics and systematics. Annu. Rev. Ecol. Systemat..

[20] Schroth W, Jarms G, Streit B, Schierwater B (2002). Speciation and phylogeography in the cosmopolitan marine moon jelly, *Aurelia* sp. BMC Evol. Biol..

[21] Carreras C (2017). Population genomics of an endemic Mediterranean fish: Differentiation by fine scale dispersal and adaptation. Sci. Rep..

[22] Riesgo A (2016). Population structure and connectivity in the Mediterranean sponge *Ircinia fasciculata* are affected by mass mortalities and hybridization. Heredity.

[23] Riesgo A (2019). Genetic diversity, connectivity and gene flow along the distribution of the emblematic Atlanto-Mediterranean sponge *Petrosia ficiformis* (Haplosclerida, Demospongiae). BMC Evol. Biol..

[24] Torrado H, Carreras C, Raventos N, Macpherson E, Pascual M (2020). Individual-based population genomics reveal different drivers of adaptation in sympatric fish. Sci. Rep..

[25] Geraldi NR (2017). Aggregations of brittle stars can perform similar ecological roles as mussel reefs. Mar. Ecol. Prog. Ser..

[26] Ravelo AM, Konar B, Bluhm BA (2015). Spatial variability of epibenthic communities on the Alaska Beaufort Shelf. Polar Biol..

[27] Stöhr S, O'Hara TD, Thuy B (2012). Global diversity of brittle stars (Echinodermata: Ophiuroidea). PLoS ONE.

[28] Boissin E, Egea E, Féral J-P, Chenuil A (2015). Contrasting population genetic structures in *Amphipholis squamata*, a complex of brooding, self-reproducing sister species sharing life history traits. Mar. Ecol. Prog. Ser..

[29] Boissin E, Feral JP, Chenuil A (2008). Defining reproductively isolated units in a cryptic and syntopic species complex using mitochondrial and nuclear markers: The brooding brittle star, *Amphipholis squamata* (Ophiuroidea). Mol. Ecol..

[30] Boissin E, Müller WEG, Chenuil A (2011). Did vicariance and adaptation drive cryptic speciation and evolution of brooding in *Ophioderma longicauda* (Echinodermata: Ophiuroidea), a common Atlanto-Mediterranean ophiuroid?. Mol. Ecol..

[31] Pérez-Portela R, Almada V, Turon X (2013). Cryptic speciation and genetic structure of widely distributed brittle stars (Ophiuroidea) in Europe. Zoolog. Scr..

[32] Humara-Gil KJ, Granja-Fernández R, Bautista-Guerrero E, Rodríguez-Troncoso AP (2022). Overlooked for over a century: *Ophioderma occultum* sp. nov. (Echinodermata), a new species of brittle star from the Eastern Pacific. J. Nat. Hist..

[33] Weber AA-T, Stöhr S, Chenuil A (2014). Genetic data, reproduction season and reproductive strategy data support the existence of biological species in *Ophioderma longicauda*. C.R. Biol..

[34] Weber AA-T, Stöhr S, Chenuil A (2019). Species delimitation in the presence of strong incomplete lineage sorting and hybridization: Lessons from *Ophioderma* (Ophiuroidea: Echinodermata). Mol. Phylogenet. Evol..

[35] Stöhr S, Weber AA-T, Boissin E, Chenuil A (2020). Resolving the *Ophioderma longicauda* (Echinodermata: Ophiuroidea) cryptic species complex: Five sisters, three of them new. Eur. J. Taxon..

[36] Sponer R, Roy MS (2002). Phylogeographic analysis of the brooding brittle star *Amphipholis squamata* (echinodermata) along the coast of new zealand reveals high cryptic genetic variation and cryptic dispersal potential. Evolution.

[37] Tortonese, E. *Fauna d'Italia: Echinodermata.* Accademia nazionale italiana di entomologia & Unione zoologica italiana (ed. Calderini) 1–422 (Bologna, 1965).

[38] Ellegren H (2014). Genome sequencing and population genomics in non-model organisms. Trends Ecol. Evol..

[39] Formenti G (2022). The era of reference genomes in conservation genomics. Trends Ecol. Evol..

[40] Catchen JM (2017). Unbroken: RADseq remains a powerful tool for understanding the genetics of adaptation in natural populations. Mol. Ecol. Resour..

[41] Leiva C (2019). Population substructure and signals of divergent adaptive selection despite admixture in the sponge *Dendrilla antarctica* from shallow waters surrounding the Antarctic Peninsula. Mol. Ecol..

[42] Saenz-Agudelo P (2022). Population genomic analyses reveal hybridization and marked differences in genetic structure of *Scurria* limpet sister species with parapatric distributions across the South Eastern Pacific. Ecol. Evol..

[43] Galaska MP, Sands CJ, Santos SR, Mahon AR, Halanych KM (2017). Crossing the divide: Admixture across the Antarctic polar front revealed by the brittle star *Astrotoma agassizii*. Biol. Bull..

[44] Galaska MP, Sands CJ, Santos SR, Mahon AR, Halanych KM (2017). Geographic structure in the Southern Ocean circumpolar brittle star *Ophionotus victoriae* (Ophiuridae) revealed from mt DNA and single**-**nucleotide polymorphism data. Ecol. Evol..

[45] Lau SCY, Strugnell JM, Sands CJ, Silva CNS, Wilson NG (2023). Genomic insights of evolutionary divergence and life history innovations in Antarctic brittle stars. Mol. Ecol..

[46] Fontaine A (1965). The feeding mechanisms of the ophiuroid *Ophiocomina nigra*. J. Mar. Biol. Assoc. U.K..

[47] Wilson J, Holme N, Barrett R (1977). Population dispersal in the brittle-star *Ophiocomina nigra* (Abildgaard)(Echinodermata: Ophiuroidea). J. Mar. Biol. Assoc. U.K..

[48] Jones A, Mallefet J (2012). Study of the luminescence in the black brittle-star *Ophiocomina nigra*: Toward a new pattern of light emission in ophiuroids. Zoosymposia.

[49] Ball B, Jangoux M (1996). The secretory system of the spines of *Ophiocomina nigra* (Echinodermata, Ophiuroidea). J. Mar. Biol. Assoc. U.K..

[50] Fontaine A (1964). The integumentary mucous secretions of the ophiuroid *Ophiocomina nigra*. J. Mar. Biol. Assoc. U.K..

[51] Hughes, D. Subtidal brittlestar beds (volume IV). *An overview of dynamics and sensitivity characteristics for conservation management of marine SACs. Scottish Association for Marine Science (UK Marine SACs Project)* (1998).

[52] Fontaine A (1962). The colours of *Ophiocomina nigra* (Abildgaard): I colour variation and its relation to distribution. J. Mar. Biol. Assoc. U.K..

[53] Mortensen, T. Handbook of the Echinoderms of the British Isles. (1927).

[54] Fontaine A (1962). The colours of *Ophiocomina nigra* (Abildgaard): II. The occurrence of melanin and fluorescent pigments. J. Mar. Biol. Assoc. U.K..

[55] Pritchard JK, Stephens M, Donnelly P (2000). Inference of population structure using multilocus genotype data. Genetics.

[56] Jombart T, Devillard S, Balloux F (2010). Discriminant analysis of principal components: A new method for the analysis of genetically structured populations. BMC Genet..

[57] Liu X, Fu Y-X (2020). Stairway Plot 2: Demographic history inference with folded SNP frequency spectra. Genome Biol..

[58] Fox DL (1976). Animal Biochromes and Structural Colours: Physical, Chemical, Distributional & Physiological Features of Coloured Bodies in the Animal World.

[59] Borrero-Pérez G, González-Wangüemert M, Marcos C, Pérez-Ruzafa A (2011). Phylogeography of the Atlanto**-**Mediterranean sea cucumber *Holothuria* (Holothuria) *mammata*: The combined effects of historical processes and current oceanographical pattern. Mol. Ecol..

[60] Siegel D (2008). The stochastic nature of larval connectivity among nearshore marine populations. Proc. Natl. Acad. Sci..

[61] Jenkins TL, Castilho R, Stevens JR (2018). Meta-analysis of northeast Atlantic marine taxa shows contrasting phylogeographic patterns following post-LGM expansions. PeerJ.

[62] Osman MB (2021). Globally resolved surface temperatures since the Last Glacial Maximum. Nature.

[63] Kimura M, Ohta T (1969). The average number of generations until fixation of a mutant gene in a finite population. Genetics.

[64] Tiffin P, Ross-Ibarra J (2014). Advances and limits of using population genetics to understand local adaptation. Trends Ecol. Evol..

[65] Hall TA (1999). BioEdit: A user-friendly biological sequence alignment editor and analysis program for Windows 95/98/NT. Nucleic Acids Symp. Ser..

[66] Tamura K, Stecher G, Peterson D, Filipski A, Kumar S (2013). MEGA6: Molecular evolutionary genetics analysis version 6.0. Mol. Biol. Evol..

[67] Peterson BK, Weber JN, Kay EH, Fisher HS, Hoekstra HE (2012). Double digest RADseq: An inexpensive method for de novo SNP discovery and genotyping in model and non-model species. PLoS ONE.

[68] Doyle JJ, Doyle JL (1987). A rapid DNA isolation procedure for small quantities of fresh leaf tissue. Phytochem. Bull..

[69] Catchen J, Hohenlohe PA, Bassham S, Amores A, Cresko WA (2013). Stacks: An analysis tool set for population genomics. Mol. Ecol..

[70] Jeffries DL (2016). Comparing RAD seq and microsatellites to infer complex phylogeographic patterns, an empirical perspective in the Crucian carp, *Carassius carassius* L. Mol. Ecol..

[71] Paris JR, Stevens JR, Catchen JM (2017). Lost in parameter space: A road map for stacks. Methods Ecol. Evol..

[72] Jombart T (2008). adegemet: A R package for the multivariate analysis of genetic markers. Bioinformatics.

[73] Puritz JB, Hollenbeck CM, Gold JR (2014). dDocent: A RADseq, variant-calling pipeline designed for population genomics of non-model organisms. PeerJ.

[74] Excoffier L, Lischer HE (2010). Arlequin suite ver 3.5: A new series of programs to perform population genetics analyses under Linux and Windows. Mol. Ecol. Resourc..

[75] Foll M, Gaggiotti O (2008). A genome-scan method to identify selected loci appropriate for both dominant and codominant markers: A Bayesian perspective. Genetics.

[76] Librado P, Rozas J (2009). DnaSP v5: A software for comprehensive analysis of DNA polymorphism data. Bioinformatics.

[77] Leigh JW, Bryant D (2015). POPART: Full-feature software for haplotype network construction. Methods Ecol. Evol..

[78] Clement M, Posada D, Crandall KA (2000). TCS: A computer program to estimate gene genealogies. Mol. Ecol..

[79] Benjamini Y, Yekutieli D (2001). The control of the false discovery rate in multiple testing under dependency. Ann. Stat..

[80] Meirmans PG (2020). genodive version 3.0: Easy-to-use software for the analysis of genetic data of diploids and polyploids. Mol. Ecol. Resourc..

[81] Earl DA, von Holdt BM (2012). STRUCTURE HARVESTER: A website and program for visualizing STRUCTURE output and implementing the Evanno method. Conserv. Genet. Resourc..

[82] Kopelman NM, Mayzel J, Jakobsson M, Rosenberg NA, Mayrose I (2015). Clumpak: A program for identifying clustering modes and packaging population structure inferences across K. Mol. Ecol. Resour..

[83] Keenan K, McGinnity P, Cross TF, Crozier WW, Prodöhl PA (2013). diveRsity: An R package for the estimation and exploration of population genetics parameters and their associated errors. Methods Ecol. Evol..

[84] Sundqvist L, Keenan K, Zackrisson M, Prodöhl P, Kleinhans D (2016). Directional genetic differentiation and relative migration. Ecol. Evol..

[85] Rozas J (2017). DnaSP 6: DNA sequence polymorphism analysis of large data sets. Mol. Biol. Evol..

[86] Suchard MA (2018). Bayesian phylogenetic and phylodynamic data integration using BEAST 1.10. Virus Evol..

[87] Rogers AR, Harpending H (1992). Population growth makes waves in the distribution of pairwise genetic differences. Mol. Biol. Evol..

[88] Ramos-Onsins SE, Rozas J (2002). Statistical properties of new neutrality tests against population growth. Mol. Biol. Evol..

[89] Posada D (2008). jModelTest: Phylogenetic model averaging. Mol. Biol. Evol..

[90] Bribiesca-Contreras G, Verbruggen H, Hugall AF, O’Hara TD (2019). Global biogeographic structuring of tropical shallow-water brittle stars. J. Biogeogr..

[91] Rambaut A, Drummond AJ, Xie D, Baele G, Suchard MA (2018). Posterior summarization in Bayesian phylogenetics using Tracer 1.7. Systemat. Biol..

[92] Yuasa H (2021). Elucidation of the speciation history of three sister species of crown-of-thorns starfish (*Acanthaster* spp.) based on genomic analysis. DNA Res..

[93] Wickham H (2016). ggplot2: Elegant Graphics for Data Analysis.

[94] QGIS.org. QGIS Geographic Information System. QGIS Association. http://www.qgis.org (2023).

