# Supplementary material for: Exceptional population genomic homogeneity in the black brittle star Ophiocomina nigra (Ophiuroidea, Echinodermata) along the Atlantic-Mediterranean coast
Source: Sci Rep. 2023 Jul 31;13:12349. doi: 10.1038/s41598-023-39584-7 (PMC10390532; doi:10.1038/s41598-023-39584-7)
Supplement: Supplementary file 1 — Supplementary Information. [file 41598_2023_39584_MOESM1_ESM.pdf]

SUPPLEMENTARY MATERIAL

Exceptional population genomic homogeneity in the black brittle star *Ophiocomina nigra* (Ophiuroidea, Echinodermata) along the Atlantic-Mediterranean coast

Carlos Leiva, Laia Pérez-Sorribes, Sara González-Delgado, Sandra Ortiz, Owen S. Wangensteen, Rocío Pérez-Portela

	CAI	STF	BLA	HER	CNE	TAR	ROS	KRI
**CAI**	-	-0.019	-0.017	-0.024	0.018	-0.008	-0.022	0.015
**STF**	-0.150	-	-0.012	-0.013	0.029	0.006	-0.012	0.011
**BLA**	-0.113	-0.030	-	-0.024	0.024	0.018	-0.024	0.030
**HER**	-0.173	-0.008	0.019	-	0.006	0.014	-0.004	0.032
**CNE**	0.066	0.034	-0.005	0.168*	-	0.009	-0.019	0.018
**TAR**	-0.125	-0.031	-0.008	-0.028	0.150*	-	-0.001	-0.024
**ROS**	-0.096	-0.023	-0.036	0.045	-0.033	0.016	-	0.005
**KRI**	-0.067	-0.011	-0.039	0.111	-0.078	0.076	-0.059	-

SM1. Pairwise distances (F_{ST}) between sampling sites from: the *COI* (above the diagonal) and ddRADseq-derived SNPs (below the diagonal). *Significant *p*-adjusted ≤0.05.

Source of variation	d.f	Sum of squares	% variance	p-value	Fixation index
**Between morphotypes**	1	4.35	-0.66	0.639±0.004	-6.64×10^{-3}
**Among localities between morphotypes**	8	50.76	0.46	0.310±0.004	4.54×10^{-3}
**Between geographical regions**	2	8.15	-0.84	0.773±0.001	-8.42x10-^{3}
**Among localities among geographical regions**	7	46.96	0.85	0.227±0.001	8.46x10-^{3}
**Inside localities**	182	1065.68	100.21	0.365±0.005	-2.06×10^{-3}
**TOTAL**	191	1120.79			

SM2. Analysis of molecular variance (AMOVA) from the *COI* fragment including two different grouping of the sampling localities: based on morphotypes (A and B described by Tortonese, 1965), and based on geographical area of collection (NW Mediterranean, Atlantic Ocean and Alboran Sea).

Source of variation	d.f	Sum of squares	% variance	p-value	Fixation index
**Between morphotypes**	1	0.035	-0.67	0.429±0.004	-6.72x10^{-3}
**Among localities between morphotypes**	6	0.381	0.10	0.342±0.005	9.5x10^{-4}
**Between geographical regions**	2	0.111	-0.14	0.460±0.005	-1.39x10^{-3}
**Among localities among geographical regions**	5	0.306	-0.05	0.395±0.005	4.6x10^{-4}
**Inside localities**	210	13.001	100.58	0.389±0.005	-6.72x10^{-3}
**TOTAL**	217	13.417			

SM3. Analysis of molecular variance (AMOVA) from the ddRADseq-derived SNPs including two different grouping of the sampling localities: based on morphotypes (A and B described by Tortonese, 1965), and based on geographical area of collection (NW Mediterranean, Atlantic Ocean and Alboran Sea).

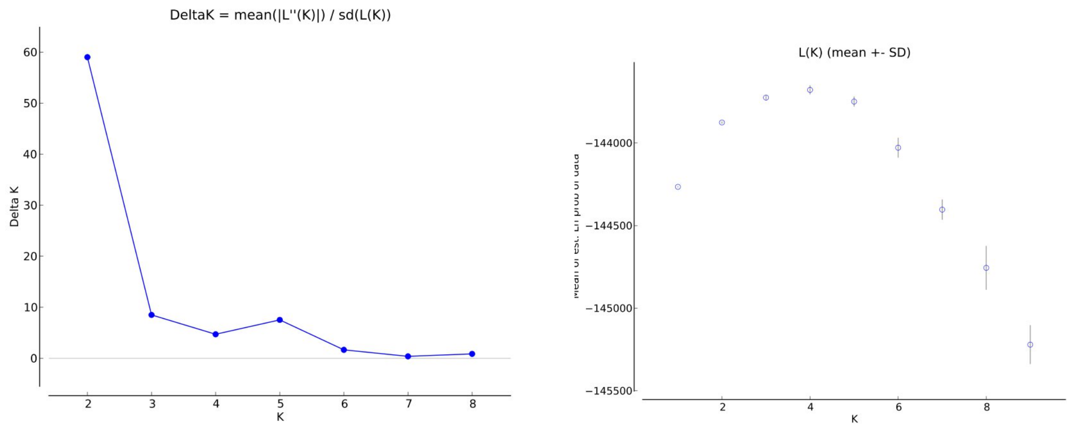

SM4. Delta K plot (left) and Ln of the posterior probabilities (right) of each K from the STRUCTURE Bayesian clustering analysis.

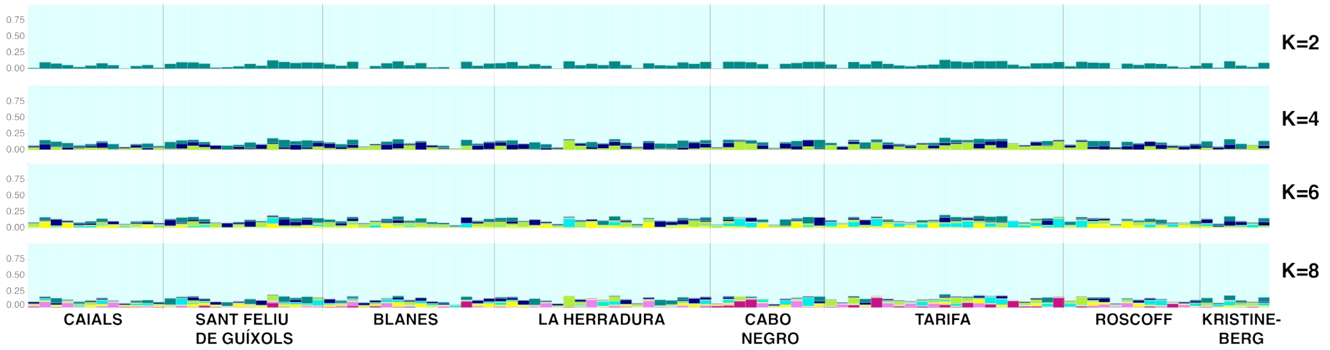

SM5. STRUCTURE barplot outputs of *Ophiocomina nigra* from the ddRADseq-derived SNPs. Posterior probabilities of individual assignment with $K = 2, 4, 6$ and 8. Different clusters are represented by different colours.

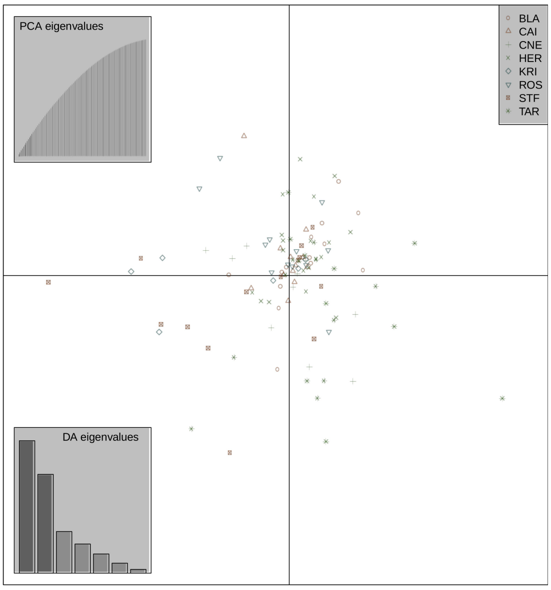

SM6. DAPC result from the ddRADseq-derived SNPs and based on the genetic cluster detected. The different point-patterns and colours represent the sampling localities. Since only one main cluster is detected, no grouping of individuals per sampling locality is observed. The PCA and DA sub-graphs represent the number of principal components and discriminant functions retained for the analysis, respectively

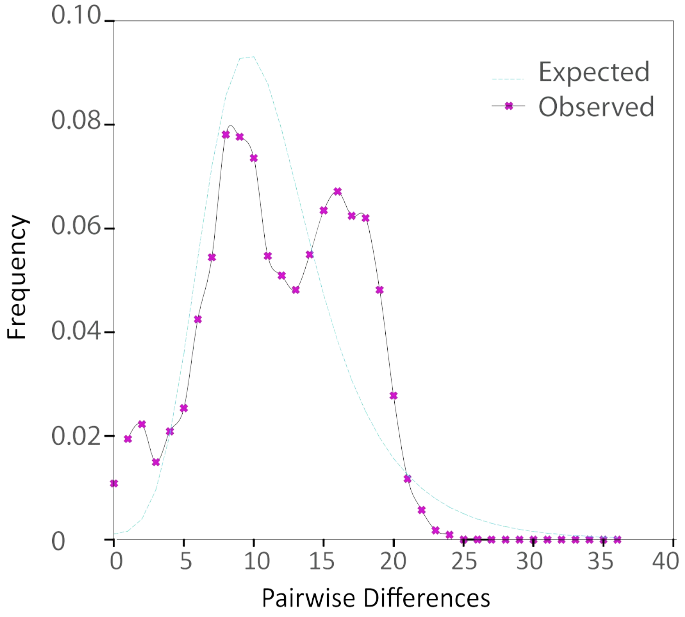

SM7. The Mismatch distribution of *O. nigra* from the *COI* fragment.

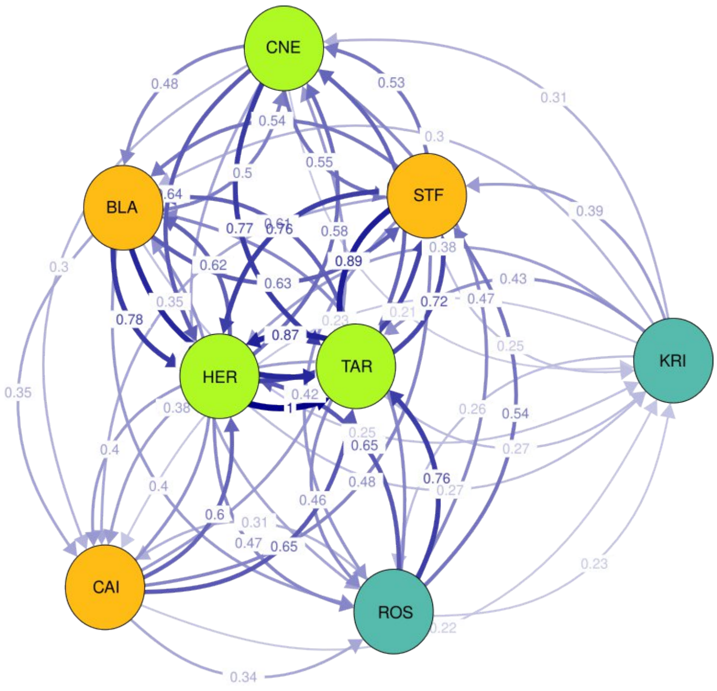

SM8. Complete G_{ST} migration network among sampling sites in *O. nigra*. The circles represent the sampling sites and the numbers on the arrows show the relative migration among sampling sites.
